# Charge Carrier Scattering in Polymers: A New Neutral Coupled Soliton Channel

**DOI:** 10.1038/s41598-018-24948-1

**Published:** 2018-04-26

**Authors:** Luiz Antonio Ribeiro, Fábio Ferreira Monteiro, Wiliam Ferreira da Cunha, Geraldo Magela e Silva

**Affiliations:** 1 Department of Physics, Chemistry and Biology, Linköping University, SE-58183 Linköping, Sweden; 2 International Center for Condensed Matter Physics, University of Brasília, P.O. Box 04531, 70.919-970 Brasília, DF Brazil; 3 Quantum Theory Project, University of Florida, Gainesville, Florida 32611-2085 USA; 4 Institute of Physics, University of Brasilia, 70.919-970 Brasilia, Brazil

## Abstract

The dynamical scattering of two oppositely charged bipolarons in non-degenerate organic semiconducting lattices is numerically investigated in the framework of a one-dimensional tight-biding–Hubbard model that includes lattice relaxation. Our findings show that it is possible for the bipolaron pair to merge into a state composed of a confined soliton-antisoliton pair, which is characterized by the appearance of states within less than 0.1 eV from the Fermi level. This compound is in a narrow analogy to a meson confining a quark-antiquark pair. Interestingly, solitons are quasi-particles theoretically predicted to arise only in polymer lattices with degenerate ground state: in the general case of non-degenerate ground state polymers, isolated solitons are not allowed.

## Introduction

Luminescent polymers have been considered the most promising class of materials in developing new technologies for light-emitting devices since the discovery of Poly (p-phenylenevinylene) (PPV) and its remarkable electroluminescence properties^1–3^. Indeed, PPV (or PPV-like) systems have been successfully employed as active components in developing polymer light-emitting diodes (PLEDs) with high quantum yield of luminescence^4,5^ and low environmental impact. The outstanding performance of these materials follows from the unusual nature of the structures involved in the charge transport as well as their interaction. Hence, the exceptional behavior of such structures ought to be carefully investigated, and this includes the analysis of different particles. It is now accepted that both the quasiparticle and the electron-hole transport picture are to be considered in order to provide a correct description of the mechanisms involved in these materials^6^. For instance, in a PLED, the mechanism of charge recombination and subsequent formation of excited species is one of the key steps behind its operation. Such recombination is generally accomplished by a scattering process between quasi-particles.

When an electron or hole is injected into a polymer lattice, a self-localized composite state containing the charge surrounded by a cloud of phonons is formed due to the strong electron-lattice interaction presented by these materials. Such state is identified as a quasi-particle called polaron. It possesses charge ±*e* and spin ±1/2. In situations where the process of charge injection leads to a large concentration of polarons, a pair of acoustic polarons, with same charge and antiparallel spins, can merge into an acoustic bipolaron. This kind of quasi-particle, in turn, is spinless and present charge ±3*e*. It is already well accepted that an electron-bipolaron (negatively charged particle) and a hole-bipolaron (positively charged particle) may recombine to form a mixed state composed of bipolarons and excitons^7–10^. The light emission phenomenon results from the radiative decay of this kind of excited particle. Therefore, the yield in forming such species determines the electroluminescence efficiency in conjugated polymers. In an unprecedented fashion, the present study shows that two oppositely charged bipolarons can recombine into a single excited species, which is composed by a pair of coupled confined solitons. As solitons are quasi-particles theoretically expected to take place only in polymer lattices with degenerate ground state, we have determined particular excitation regimes that give rise to unreported results. Importantly, this new evidence draws attention to the different nature of excited species that play a role in the light emission in conducting polymers, which is still not completely understood. Moreover, as will be further discussed, the nature of one of these products corresponds, in many aspects, to that of mesonic systems. This fact can be explored to a better understanding and simulation of the dynamics of these subatomic particles.

It was both theoretically^11^ and experimentally^12^ demonstrated by Kobayashi and coworkers that a *trans*-polyacetylene (*t*-PA) lattice (with degenerate ground state) has a nonlinear excitation of soliton upon photon absorption due to the electron-lattice interactions. The excess of energy related to the photogenerated electron-hole pair that evolves into a soliton-antisoliton pair forms breather-like oscillations, which are characterized by collective stretching vibration of the carbon-carbon bonds^11^. Their study was the first experimental evidence of the formation and decay of a dynamical bound state formed by a soliton pair, which is created in *t*-PA after photoexcitation^12^. It should be noted that the soliton pair described in their work was not confined. From a theoretical point of view, Tretiak, Bishop, and colleagues have numerically studied the existence of photoexcited breathers in several types of conducting polymers^13,14^. Their results show that a strong breather mode is observed in a *cis*-PA lattice as a consequence of interactions between vibrations within the same phonon band^14^. Moreover, weaker breathers in PPV and polyfluorene appear due to the interaction between vibrations among several different phonon bands^13^. However, there is no evidences concerning the nature of the quasi-particles involved in the breather formation process. In spite of several theoretical predictions about the formation and decay of breather states in conducting polymers^15–20^, no study revealing the conversion of oppositely charged bipolarons into a soliton-antisoliton pair, considering non-degenerate ground state lattices, has emerged so far.

In this paper, the dynamical recombination of two oppositely charged bipolarons in conjugated polymers is numerically investigated by employing a one-dimensional Hamiltonian that considers electron-phonon and electron-electron interactions. Undoubtedly, the search for signatures of new excited species – followed by a deep understanding of the mechanisms involved in the process of their formation – may provide guidance for improving the quantum electroluminescence yields in organic semiconducting materials and is, therefore, precisely the aim of the present study. Incidentally, we provide a testing bed for meson like dynamics in a solid state system.

## Methods

The one-dimensional model employed to study the dynamical recombination of the bipolaron pair is a combined version of the Su-Schrieffer-Heeger (SSH) and Hubbard Hamiltonians^21–23^. In this way, the overall model Hamiltonian (*H*) has the form: *H* = *H*_1_ = +*H*_2_, where *H*_1_ denotes the electronic and the lattice contributions whereas *H*_2_ addresses Hubbard terms introduced to account the electron-electron interactions. The *H*_1_ contribution to the total Hamiltonian can be written in the form

1 $${H}_{1}=-\sum _{n,s}({t}_{n,n+1}{C}_{n+\mathrm{1,}s}^{\dagger }{C}_{n,s}+h\mathrm{.}c\mathrm{.})+\frac{K}{2}\sum _{n}{y}_{n}^{2}+\frac{1}{2M}\sum _{n}{p}_{n}^{2},$$

where *n* indexes the lattice sites. The operator ${C}_{n,s}^{\dagger }$ (${C}_{n,s}$) creates (annihilates) a *π*-electron with spin *s* at the *n*−th site. ${t}_{n,n+1}$ describes the hopping integral of a *π*-electron, with spin *s*, between nearest-neighboring sites and can be expressed as

2 $${t}_{n,n+1}={e}^{-i\gamma A(t)}\{[1+{(-\mathrm{1)}}^{n}{\delta }_{0}]({t}_{0}-\alpha {y}_{n})\}\mathrm{.}$$

In this hopping term, *A*(*t*) is the time-dependent vector potential that is included to account for the field induced dynamics of charge carriers and is related to the electric field by **E** = −(1/*c*)**A**. In Eq. 2, $\gamma \equiv ea/(\hslash c)$, where *e* is the absolute electronic charge, *a* is the lattice constant, and *c* is the speed of light. *t*_0_ is the strength of the hopping term for *π*-electrons with spin *s* in undimerized lattices, *α* is the electron-phonon coupling constant, and *y*_*n*_ denotes the relative displacements for lattice sites. *δ*_0_ is the Brazovskii-Kirova symmetry-breaking term^24^, which is considered here in order to model non-degenerated polymer lattices. The lattice backbone is addressed in a harmonic approximation by using the second and third parts of Eq. 1, where *K* denotes the force constant that describes the C-C σ bond, and *M* expresses the mass of a single site. Finally, the last term of the overall Hamiltonian (Hubbard term), *H*_2_, denotes the contribution of the electron-electron interactions and can be written in the following form

3 $${H}_{2}=U\sum _{i}({C}_{i,\uparrow }^{\dagger }{C}_{i,\uparrow }-\frac{1}{2})({C}_{i,\downarrow }^{\dagger }{C}_{i,\downarrow }-\frac{1}{2})+V\sum _{i}({n}_{i}-\mathrm{1)(}{n}_{i+1}-\mathrm{1).}$$

In the equation above, ${n}_{i}={C}_{i,\uparrow }^{\dagger }{C}_{i,\uparrow }+{C}_{i,\downarrow }^{\dagger }{C}_{i,\downarrow }$. *U* is the on-site and *V* the nearest-neighbor site Coulomb repulsion strength, respectively. The solution of the problem is numerically carried out according to the procedure specified in a previous work through a Hartree-Fock approximation^23^.

It is worthwhile to highlight that the initial arrangement for the lattice contains an electron-bipolaron and a hole-bipolaron. Such physical picture is achieved by employing the self-consistent procedure described in a previous work^25^. In the formation processes of these charge carriers, two electrons are added (electron-bipolaron) or removed (hole-bipolaron) from the polymer lattice generating distortions in its conformation and displacement of energy levels inside the band gap. For the case studied here, the energy levels associated to the hole-bipolaron – the Highest Occupied Molecular Orbital (HOMO) and the second Lowest Unoccupied Molecular Orbital (LUMO+1) – are both empty, thus yielding a +2*e* charge. On the other hand, the electron-bipolaron is represented by the doubly occupied levels HOMO−1 and LUMO, thus resulting in a −2*e* charge. Straightforwardly, one can realize that the initial lattice is neutral. Once obtained the initial arrangement for the lattice, the system is evolved in time by using Coupled Dynamics, as established in ref.^25^. The set of parameters employed in the model Hamiltonian used here is: *t*_0_ = 2.5 eV, *M* = 1349.14 eV × fs^2^/Å^2^, *K* = 21 eV/Å^2^, *V* = *U*/3, *δ*_0_ = 0.005 eV, and *a* = 1.22 Å. The electron-phonon coupling *α*, the on-site electron-electron interactions *U*, and the electric field *E*_0_ vary within the intervals [4.0, 6.0] eV/Å (with step-size of 0.2), [0, 1.5] eV (with step-size of 0.25), and [0.5, 2.5] mV/Å (with step-size of 0.5), respectively. These parameters were successfully used in other studies^23,26–30^.

## Results and Discussion

As pointed out above, the motivation of this work is powered by the aim of obtaining unconventional excited species that might be present in conducting polymers upon charge recombination. Alongside the new excited species yielded in the present study, other kind of beasts, such as excitons and biexcitons, were also obtained. As these later ones were already observed in other theoretical studies reported in literature^7,8,31–35^, their behavior, although of importance, will not be focus of our attention here in favor of discussing the previously unreported channel. In the numerical realizations performed in the present work, the new found product is noted to take place for *U*, *α*, and *E*_0_ ranging between 1.0–4.0 eV, 4.6–5.6 eV/Å, and 1.0–1.5 mV/Å, respectively. Once the products yielded within these intervals are qualitatively similar, we restrict our discussion to the case with *U* = 2.0 eV, *α* = 5.4 eV/Å, and *E*_0_ = 1.5 mV/Å. The fact that a reasonable span of parameters give rise to products of this unprecedentedly reported nature justify the importance of our finding: it increases the possibility of synthesis of polymers whose properties are accurately described by a given combination of parameters within this range.

**Figure 1:**
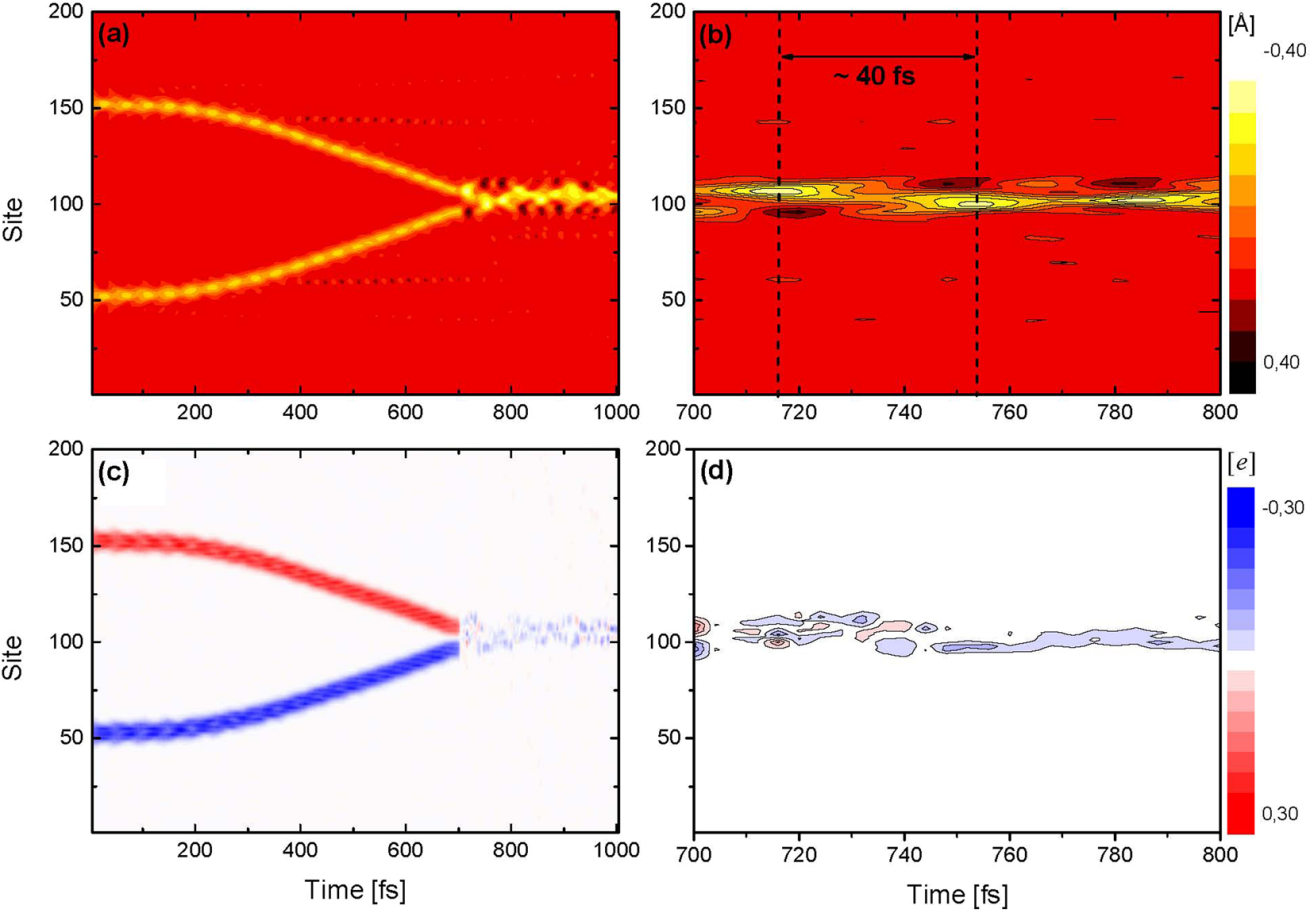
Time evolution of the staggered bond-length (**a**,**b**) and mean charge density (**c**,**d**) for the recombination dynamics of two oppositely charged bipolarons in a 200-site lattice. The right-sided panels zoom in the left-sided panels in the period between 700–800 fs.

Figure 1 displays the time evolution for the recombination dynamics between the hole- and electron-bipolaron in a 200-site lattice with periodic boundary conditions. In order to provide a clear description of our results, Fig. 1(a) and (b) present the staggered bond length, whereas Fig. 1(c) and (d), the mean charge density. These quantities are calculated according to ref.^23^. For simulation purposes, the bipolarons are initially positioned far apart from each other so that a premature interaction between them is avoided: the negative bipolaron is placed at the 50th site, whereas the positively charged one is located at the 150th site. From Fig. 1(a) one can realize that, during the first 100 fs, the strength of the electric field is turned on quasi-adiabatically – following the procedure established in refs^36,37^ – to preserve numerical stability. After this transient period, the bipolarons are accelerated and reach their saturation velocity at around 400 fs. As a straightforward consequence of reaching the saturation velocity, the energy excess imposed to the bipolarons by the external electric field is scattered in the form of phonons that propagate throughout the lattice, so that the carriers are allowed to move linearly until approximately 700 fs. At this instant the collision between the quasi-particles takes place, a process after which the original quasi-particles are observed to merge into a single new structure. This recombination mechanism yields a neutral species, which deforms the lattice in a deeper fashion when compared to the lattice deformations associated to each of the bipolarons, as can be inferred from Fig. 1(a) and (b). From Fig. 1(c), one can see that the net charge coupled to the hole-bipolaron (red contour) and to the electron-bipolaron (blue contour) vanish after the scattering process (at approximately 700 fs). Note that a very small portion of charge remains trapped to the new lattice structure generated after the recombination between the charge carriers. Naturally, such small charge is not able to transport the yielded species along the lattice. Therefore, the structure generated after the recombination mechanism is neutral. Remarkably, one can observe, from Fig. 1(a), that the recombination process yields a structure that has its deformation amplitude oscillating in time. After the collision between the bipolarons, new phonon modes are generated in the lattice. The nonlinear mixing of two or more of those vibrational modes, which are strongly coupled to the electronic degrees of freedom, leads to the formation of a breather-like state. Such state is formed due to the energy excess generated in the colision moment that induces the collective carbon-carbon oscillation and electronic exitations by means of the electron-phonon coupling term. This breather state is noted to take place only in cases where the phonon modes are non-adiabatically connected to electronic excited states. Interestingly, in the present case this excited state evolves into a coupled soliton-antisoliton pair. As can be seen in Fig. 1(a), the coupled soliton-antisoliton pair manages to keep its stability until the end of the simulation, i. e., for 300 fs. It was experimentally demonstrated that the lifetime of the breather in *t*-PA, a degenerate polymer, is very short and it decays into two single free solitons in less than 100 fs^12^. Since single solitons are energetically forbidden for non-degenerate polymers, the stability of the present structure is a reassuring feature. It has been known for decades that isolate solitons are natural solutions in degenerate polymers but polarons are to arise more commonly in nondegenerate ones^38^. This confinement of the solitons is a fundamental aspect of the system and stems from the non-degenerate character of the polymers described here.

The arising of this peculiar kind of excited state is specially interesting because it allow us to trace a deep analogy between solid state and elementary particles. Indeed, the species formed from the coupling between the soliton and the antisoliton is structured analogously to a meson, as the later is formed by a quark-antiquark pair. In the former, such interaction is mediated by the coupling between charge and phonon. In the later we have gluons binding the quarks together. Another aspect of this analogy is in that the constituents of neither pair are to be isolated found in nature. Color confinement prohibits quarks to be singly observed in the same way that symmetry considerations dictates that solitons are not to be freely found in non-degenerate polymers. Finally, the analogy can be extended to the very aspect of the energetic formation: the potential energy associated to either kind of pair increases linearly with the distance between their constituents. This is known to be true for the quarks^39^ and is also easily concluded for the solitons by noting that each one is associated to the change in the phase of the alternating bond pattern of the treated polymers and, since the ground state is non-degenerated, each site between the soliton-antisoliton pair adds to the total energy. As all these properties are formally the same, one can think of this kind of solid state system as an analogous model to treat meson dynamics.

The details of the breather-like state can be better analyzed by using Fig. 1(b). This figure zooms in the left-sided panel (Fig. 1(a)) after the recombination process. One can note that the positive (black contour) and negative (yellow contour) peaks, that represent the higher amplitude for the bond compression and elongation, respectively, exchange positions in time between the lattice sites 90 and 110. The first period of oscillation takes nearly 40 fs, as highlighted in Fig. 1(b). However, from the second period onward, there is a dephasing time imposed by the propagating phonons which are generated after the collision between the bipolaron pair that blueshifts the frequency of the breather state to roughly 30 fs. This physical scenario for the breather formation is in good agreement with both theoretical^11,13,14,19^ and experimental^12^ predictions obtained in photoexcited *t*-PA lattices: the modulation periods predicted in theses studies range from 30 to 50 fs. In the same fashion, from Fig. 1(d) – that zooms in the left-sided panel (Fig. 1(c)) in the period between 700–800 fs – one can confirm that the portion of charge that remains coupled to the formed structure is, indeed, substantially smaller and cannot establish a net charge for it, thus yielding a neutral excited species.

**Figure 2:**
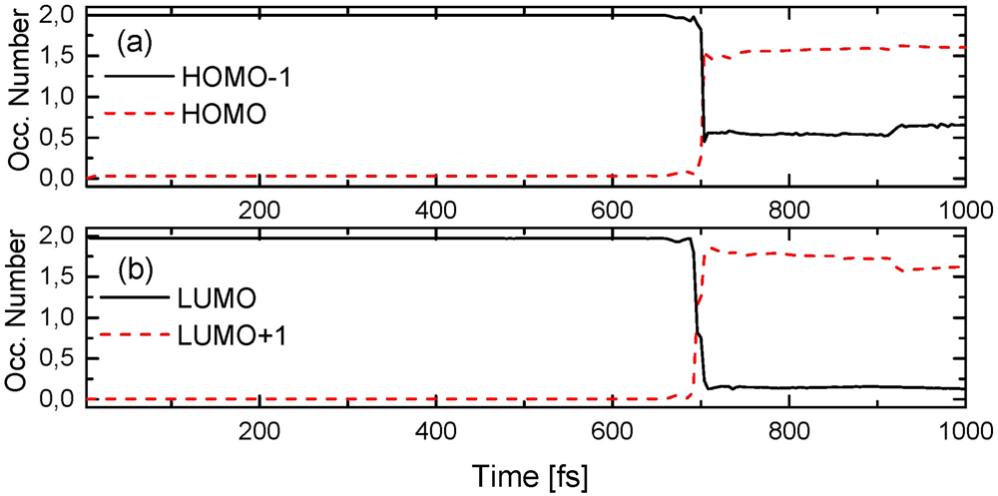
Time evolution of the occupation number for (**a**) the HOMO and (**b**) LUMO leves. In black are represented the electron-bipolaron levels HOMO−1/LUMO whereas in red are represented the hole-bipolaron levels HOMO/LUMO+1.

In order to better characterize the excited species formed after the recombination of the bipolaron pair, we present the time evolution of the intra-gap energy level occupation numbers, as shown in Fig. 2. The occupation number is derived according to ref.^25^. The combined analysis of these two measurements has proven to be an interesting manner to drawn general conclusions concerning the nature of the states yielded after the collisional processes, particularly when it comes to that of oppositely charged species^40–44^. The electron-bipolaron is a quasi-particle formed by a state of four electrons – in which two extra electrons are added to the lattice – thus producing two doubly occupied intra-gap levels. The hole-bipolaron, on the other hand, is formed by two holes, thus yielding two empty levels within the band gap. This particular arrangement for electronic occupation of the intra-gap levels is depicted in Fig. 2(a) and (b). In these figures it is possible to note that the levels HOMO−1/LUMO – the electron-bipolaron levels – are doubly occupied (black lines) whereas the HOMO/LUMO+1 levels – the hole-bipolaron levels – are both empty (red lines). During the evolution of the bipolarons towards their collision, i. e., before 700 fs, the levels evolve in time preserving their initial occupation. Immediately afterwards, the interaction between the bipolarons takes place and the occupation of the levels starts to change. From Fig. 2(a) one can realize that the occupation for the HOMO−1 level drops to nearly 0.5 whereas the occupation for the HOMO level rises to approximately 1.5. In other words, an electronic fraction (almost 1.5 electrons) is excited and then transferred from the HOMO−1 to HOMO. This electronic excitation is imposed by the energy transfer from the vibrational modes that are generated in the collision to the electronic degrees of freedom, which are strongly coupled to the lattice by means of *α*. Likewise, obeying the Hamiltonian electron-hole symmetry, the two electrons that originally occupied the LUMO level migrate to the LUMO+1 (nearly 1.5 electrons) upon excitation, and to upper levels (almost 0.5 of an electron) within the conduction band – which are not shown here – as displayed in Fig. 2(b). This new arrangement for the electronic occupation deeply places the HOMO−1/LUMO+1 levels inside the band gap, as discussed in what follows.

**Figure 3:**
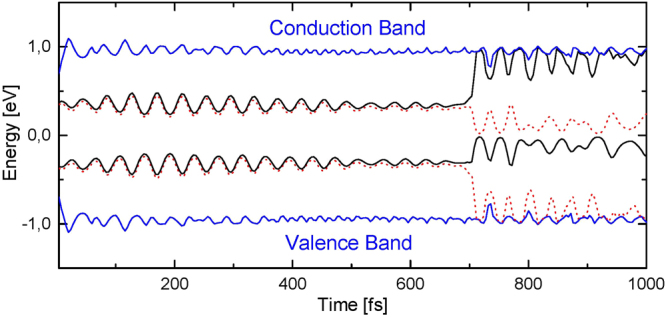
Time evolution of the intra-gap energy levels. The blue lines correspond to the last and first levels of the valence and conduction bands, respectively.

With a glimpse to Fig. 3, that depicts the time evolution of the intra-gap levels, one can easily note that, just after the bipolarons collision (at about 700 fs), and upon excitation, the almost doubly occupied HOMO level returns to the valence band whereas the closely empty LUMO level ascends to the conduction band. Concurrently, the HOMO−1/LUMO+1 levels shift upward/downward towards the center of the band gap. The symmetry degree presented by the pair of levels HOMO/LUMO and HOMO−1/LUMO+1 after the recombination process – that can be clearly noted by observing their oscillatory pattern – and the new arrangement of these levels within the band gap corroborate that the HOMO−1/LUMO+1 are the levels of a pair of solitons. The total occupation of these new intra-gap levels is close to two, where the HOMO−1 level is almost empty whereas the LUMO+1 level is occupied by approximately two electrons. This configuration for the energy levels denotes a pair of coupled solitons. A positive soliton is represented by just one empty level localized in the middle of the band, in this case, the HOMO−1 level. Conversely, the negative soliton (or antisoliton) presents only one doubly occupied level, here the LUMO+1 level, in the center of the band gap. It can be directly noticed that this configuration yields a neutral and spinless excited quasi-particle that is formed by the coupled soliton/antisoliton pair.

**Figure 4:**
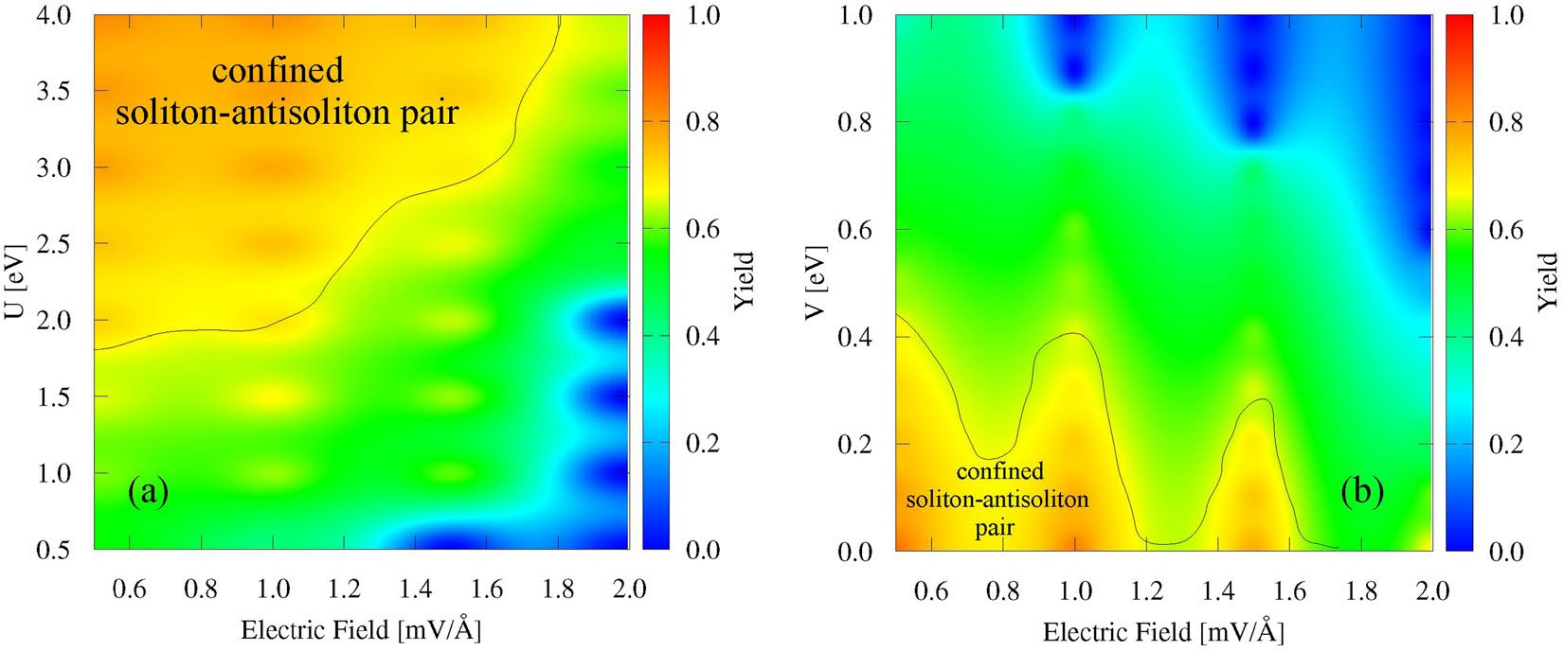
Phase diagrams of E and U (**a**); and E and V (**b**); for soliton–antisoliton yields (presented on the color pattern).

We summarize our results with an analysis of two phase diagrams relating the yield of formation of the soliton–antisoliton pair (percentuals in the the color scale) to the electric field, and the Hubbard parameters U and V. Such property is a measure of how efficient is the formation of the structure and its definition is given in the literature^25^. Figure 4(a) presents the yield of formation of the soliton–antisoliton pair as a function of the electric field and U. In this case, preliminary analysis allowed us to restrict the value of V to 0.35 eV. One can see that higher yields are localized in the upper left part of the figure. This is consistent to the spin density wave character that on site coulombic interactions are known to provide to the system. Also, note that the electric field tends to disfavor the creation of the bound pair because it provides the initial structures with such a high kinetic energy that allows them to pass one another without a reasonable time to interact. In the figure, this is the reason why high yields are localized on the left side of the panel. Combining these two effects, we conclude that the preferable region for the confined pair to take place is that of the second quadrant. Figure 4(b) presents the effects of the nearest neighbor coulomb repulsion, i.e., V. In order for our results to be representative, we adopted the limiting value of U = 4.00 eV. As V is responsible for a charge density wave character of the system, when its value is increased, it makes the initial bipolarons to move far away from each other, hindering the U effect that would otherwise allow charge transfer to couple the pair. Therefore, all else unchanged, one ends up with higher yields for smaller V. As the electric field effect is the same previously discussed, the region of highest yield is in the third quadrant of the figure.

## Conclusion

It was obtained that, upon scattering, two oppositely charged bipolarons can merge into a coupled confined soliton-antisoliton pair in non-degenerate organic semiconducting lattices. These quasi-particles are characterized by the appearence of energy levels within 0.1 eV from midgap. The obtained system is found to be the solid state analogous to a coupled quark-antiquark pair in a meson, respecting confinement, particle-antiparticle pairing, linear effective bonding potential energy, and the nonexistence of free constituints. Such unconventional result is dependent of the interplay between the parameters employed in the model Hamiltonian. As the choice of these parameters reflects values that polymers are subjected to, we have determined a new channel for the product of carrier recombination. Another feature of the scattering process is the agreement of breathers’ frequency with the experimentally expected result of 30 fs. Interestingly, though, these breathers are observed to be rather stable, as their lifetime, being greater than 300 fs, is substantially larger than what is observed in degenerate polymers. We have, therefore, found a new element acting in the quasi-particle picture of non-degenerate conducting polymers.
